# Keeping track of the growing number of biological functions of chitin and its interaction partners in biomedical research

**DOI:** 10.1093/glycob/cwv005

**Published:** 2015-01-16

**Authors:** Bjørn EV Koch, Jens Stougaard, Herman P Spaink

**Affiliations:** 2 Department of Molecular Biology and Genetics, Aarhus University, Aarhus C, Denmark; 3 Leiden University, Institute of Biology, Leiden, The Netherlands

**Keywords:** chitin, chitinases, chitinase-like proteins, immunogenicity, zebrafish

## Abstract

Chitin is a vital polysaccharide component of protective structures in many eukaryotic organisms but seems absent in vertebrates. Chitin or chitin oligomers are therefore prime candidates for non-self-molecules, which are recognized and degraded by the vertebrate immune system. Despite the absence of polymeric chitin in vertebrates, chitinases and chitinase-like proteins (CLPs) are well conserved in vertebrate species. In many studies, these proteins have been found to be involved in immune regulation and in mediating the degradation of chitinous external protective structures of invading pathogens. Several important aspects of chitin immunostimulation have recently been uncovered, advancing our understanding of the complex regulatory mechanisms that chitin mediates. Likewise, the last few years have seen large advances in our understanding of the mechanisms and molecular interactions of chitinases and CLPs in relation to immune response regulation. It is becoming increasingly clear that their function in this context is not exclusive to chitin producing pathogens, but includes bacterial infections and cancer signaling as well. Here we provide an overview of the immune signaling properties of chitin and other closely related biomolecules. We also review the latest literature on chitinases and CLPs of the GH18 family. Finally, we examine the existing literature on zebrafish chitinases, and propose the use of zebrafish as a versatile model to complement the existing murine models. This could especially be of benefit to the exploration of the function of chitinases in infectious diseases using high-throughput approaches and pharmaceutical interventions.

## Chitin

**Fig. 1.**
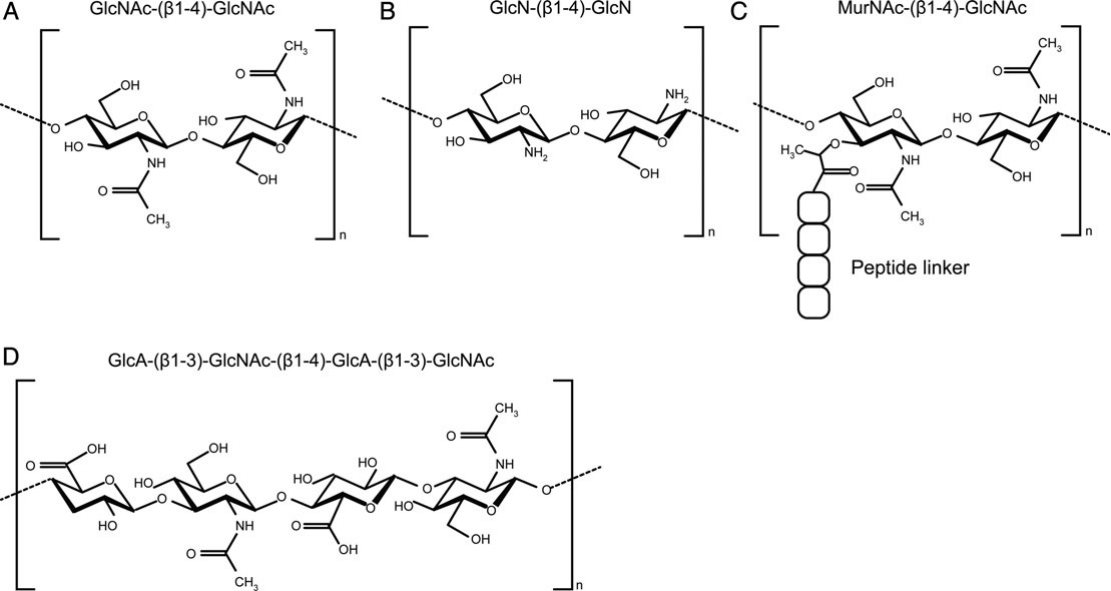
Chemical structures of various naturally occurring GlcNAc polymers. (**A**) Chitin, the homopolymeric form of β(1–4) GlcNAc residues is produced in vast quantities by all species of fungi as well as ∼90% of the species in the animal kingdom but notably not by vertebrates. (**B**) Chitosan, the heterogeneous group of fully or partially deacetylated chitin, can be purified from natural sources or produced by chemical deacetylation of chitin (Kumar et al. 2004). (**C**) Peptidoglycan, found in bacterial cell walls, is a complex structure consisting of polymers GlcNAc and N-acetylated muramic acid, linked together by β(1–4) glycosidic bonds and cross-linked by amino acid linkers. Certain chitinases hydrolyze peptidoglycan (Bokma et al. 1997). (**D**) Hyaluronic acid, a polymer of GlcNAc and glucuronic acid disaccharides linked by alternating β(1–4) and β(1–3) glycosidic bonds, is found in connective tissues of vertebrates. The zebrafish *HAS2* is able to synthesize short GlcNAc oligomers during embryo development (Semino et al. 1996). Such oligomers were proposed to function as primers for HA synthesis (Semino and Allende 2000).

Chitin is a homopolymeric form of β(1–4)-linked *N*-acetyl-d-glucosamine (GlcNAc) residues, which constitutes most of the exoskeleton of nematodes, arthropods, mollusks and the cell wall of fungi (Jeuniaux and Voss-Foucart 1991). Although the ability to synthesize GlcNAc moieties is widespread among organisms and important for protein glycosylation, vertebrates appear to have lost the enzymatic capacity to produce GlcNAc polymers like chitin and peptidoglycan, found in bacterial cell walls (Figure 1). Some GlcNAc polymers may therefore be perceived as pathogen-associated molecular patterns (PAMPs) in non-self-recognition or play a role as signal molecules when vertebrates come into contact with chitin or deacetylated chitin fractions, chitosan. In this review, we aim to provide an overview of chitin responses in vertebrates with a focus on the involvement of chitinases.

### Chitin: tissue-specific and size-dependent regulation of innate immune responses

In plants, short chitin oligomers, peptidoglycan and lipo-chito oligosaccharides of microbial origin are recognized as PAMPs and symbiotic signal molecules, respectively (Madsen et al. 2010). Specialized receptors such as the chitin elicitor receptor kinase 1 and Nod factor receptor 1 and 5 (Madsen et al. 2003; Radutoiu et al. 2003) bind these ligands and trigger downstream responses (Madsen et al. 2011; Broghammer et al. 2012). In vertebrates, which have no enzymatic capacity for chitin synthesis, chitin is also a potential PAMP. Indeed, the protective immunostimulatory effects of chitin particles against fungal infections in mice were reported almost 30 years ago, with the discovery that intraperitoneal (IP) injection of mice with chitin and chitosan reduced the mortality caused by candidiasis (Suzuki et al. 1984). Thus, the realization that chitin possesses immunostimulatory properties when introduced in vertebrates is not new, however, our understanding of the underlying molecular mechanisms is still somewhat rudimentary, and contrasting observations still have to be reconciled.

In order to study the immune-stimulating properties of chitin, particulate (as opposed to soluble) chitin fractions have been applied to murine models by different routes of exposure. A mounting body of evidence indicates that several factors, such as tissue of exposure, chitin particle size fractions and single versus repeated exposure, will profoundly influence the outcome of particulate chitin challenge.

In one such study, the immune signaling properties of chitin were assessed after delivery of chitin intranasally and intraperitoneally to mice. In both settings, an innate type 2 immune response characterized by the migration to and accumulation of eosinophils was observed. Likewise, alternative macrophage activation was found at both sites of chitin delivery. However, while intraperitoneal chitin challenge was accompanied by a transient neutrophilic response, this was not observed in the lung (Reese et al. 2007).

Several studies have provided convincing evidence for multiple different signaling pathways to account for the observed alveolar eosinophilia and alternative activation of macrophages in response to chitin challenge. Leukotriene B4 derived from macrophages was important for the eosinophilic response in the lung, mediated by signaling through the receptor BLT1 (Reese et al. 2007). Furthermore, two recently published studies implicated epithelial chitin perception in the observed effects. Chitin microparticles (CMPs) normally defined as particles of >10 μm in diameter were reported to induce alternative macrophage activation through CCL2 signaling in response to binding of chitin by airway epithelial cells (Roy et al. 2012). In addition to this, chitin induced the release of epithelial-associated cytokines interleukin-25 (IL-25), IL-33 and thymic stromal lymphopoitin (TSLP), all of which have been shown to nonredundantly activate production of the canonical type 2 cytokines IL-5 and IL-13 in innate lymphoid type 2 cells (ILC2s). This induction also led to both eosinophilia and alternative activation of macrophages (Van Dyken et al. 2014).

While the innate type 2 response observed by Reese et al. was not dependent on the myeloid differentiation primary response gene 88 (Reese et al. 2007), the common downstream adaptor for most Toll-like receptors (TLRs), other studies have provided evidence for strong TLR-2-mediated proinflammatory responses to intranasal chitin delivery. This response was characterized by a robust tissue neutrophilic response driven by induction of IL-17A, with no associated eosinophilia (Da Silva et al. 2008). These different observations were attributed to different experimental designs, specifically single versus repeated chitin challenge, and a shift to eosinophilia upon repeated chitin treatment was reported.

Intriguingly, van Dyken and coworkers, who also applied a repeated chitin exposure approach, found that genetic ablation of the ILC2s relaying the lung epithelial cytokine signaling led to a neutrophil rich inflammatory response to chitin challenge. This response was driven by activation of tissue residing γδT cells and raised expression of proinflammatory cytokines such as TNF-α, IL-1β and IL-17A (Van Dyken et al. 2014). One interpretation of these observations is that several immune cell subsets will recognize the same chitin stimulant, and interactions between the activated pathways will affect the ultimate outcome.

Other investigators, however, have attributed the discrepancies to differences in the size of chitin particles, particularly whether or not particles are of a phagocytizible size. One study found chitin fragments of ∼50 μm accountable for alternative macrophage activation and tissue eosinophilia in the peritoneum, while CMPs were found to drive classical macrophage activation and inflammation through TNF-α induction and reduction of IL-10 (Kogiso et al. 2011). To the best of our knowledge, little is known about the maximal sizes of chitin particles that can be phagocytized by immune cells. In vitro evidence exist that CMPs are in fact phagocytized (Kogiso et al. 2011). By comparison histological sections, of labeled large chitin beads (50–70 µm) in mouse lungs over a 48-h time frame, demonstrated a slow progressive particle degradation in vivo, thereby indicating that these size fractions are definitely too large to be phagocytized (Van Dyken et al. 2014). Indeed, many studies have shown that the nature of immune responses are profoundly dependent on the size of challenging chitin particles. While most studies agree that CMP challenge induces a proinflammatory TNF-α response (Shibata et al. 1997; Nishiyama et al. 2006; Da Silva et al. 2009; Kogiso et al. 2011), there are contrasting reports on the induction of anti-inflammatory IL-10. Some studies found that CMPs induce IL-10 expression (Da Silva et al. 2009) while others found that they explicitly did not induce IL-10 (Nishiyama et al. 2006; Kogiso et al. 2011). These contrasting results may arise from differences in experimental design, as IL-10 induction was found in an alveolar delivery model versus peritoneal delivery in the case of the studies reporting no IL-10 induction. In murine models of gut inflammation, CMP delivery induced IL-10 and IFN-γ, which confers marked protection against colitis (Nagatani et al. 2012). Very recently, it was reported that the concentration of the stimulant influenced the secretion of IL-10 versus TNF-α from human peripheral blood mononuclear cells. While low concentrations of chitin favored IL-10, higher concentrations led to a strong TNF-α secretion (Wagener et al. 2014).

**Fig. 2.**
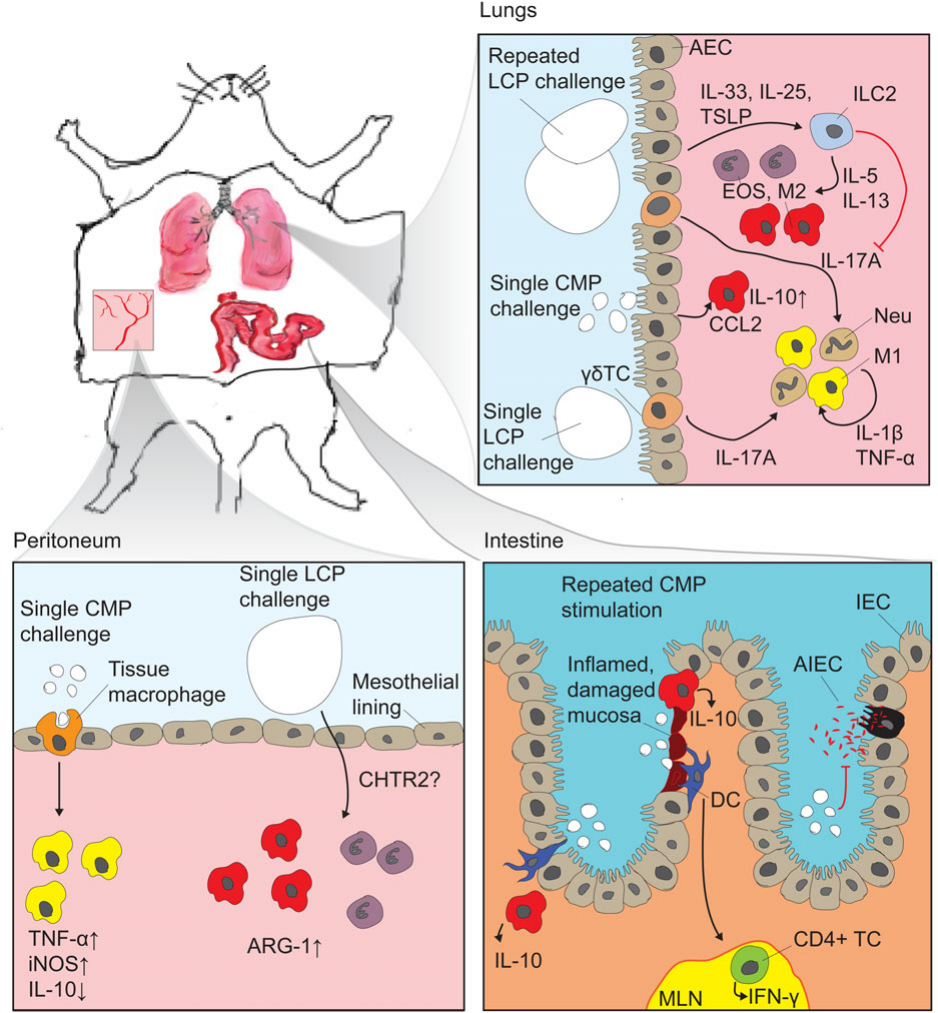
Murine in vivo observations of chitin immune-stimulating effects: The experimental approaches have generally been based on delivering chitin particles in liquid suspensions by one of the following routes: (i) intranasal or intratracheal delivery followed by broncheoalveolar lavage (BAL) analysis and histological examinations of lung tissue, (ii) IP injection followed by analysis of peritoneal lavage cell composition or (iii) gastrointestinal delivery by oral gavage followed by histological examination of mucosal barrier integrity and inflammation. In the lung, recent studies established a profound impact of epithelial derived signals, which hitherto have not been studied in other tissues. Repeated delivery with large chitin particles (LCPs) well above phagocytosable size induced IL-25, IL-33 and TSLP, which, via innate ILC2, induced type 2 innate immune responses characterized by tissue eosinophilia and alternative macrophage activation (M2). At the same time, ILC2s exerted an inhibiting effect on type 1 responses which would otherwise be driven by IL-17A released from γδ T cells (Van Dyken et al. 2014). Airway epithelial cells, upon binding of CMP, release CCL2 which further drive M2 (Roy et al. 2012), and CMP was observed to induce the production of IL-10 in BAL macrophages (Da Silva et al. 2009). Single exposure to LCPs leads to an IL-17A driven type 1 immune response which is strongly dependent on TLR-2. It is not known which cells are responsible for perceiving the chitin particles (Da Silva et al. 2008, 2009). In the peritoneum single exposure to CMP leads to phagocytosis-dependent proinflammatory signaling characterized by induction of TNF-α and inducible nitric oxide synthase while downregulating IL-10 expression. LCP induces tissue eosinophilia and M2 characterized by arginase 1 induction by a signaling pathway affected by CHTR2 (Kogiso et al. 2011). The possible signaling influence from the mesothelial cells lining the peritoneal cavity was not investigated. In murine colitis models, CMPs stimulated the accumulation of IL-10 producing cells, presumably alternatively activated macrophages, both at inflamed and non-inflamed sites. They have been shown in vitro to be internalized by dendritic cells which are then thought to stimulate IFN-γ production from CD4+ T cells (CD4+TCs) in the mesenteric lymph nodes (MLN) (Nagatani et al. 2012). Furthermore, CMPs inhibited adherence and invasion of adherent invasive *E. coli* in IECs (Kawada et al. 2008).

When considering the literature as a whole, it is clear that the overall outcome of chitin particle stimulation in terms of inflammatory responses, cellular migration and macrophage activation, is subject to different influences deriving from different cell-types, and observing only one of these factors will lead to confusion and misinterpretations. Figure 2 provides a summary of the observed immune responses to different chitin particles in different tissues.

The soluble, oligomeric forms of chitin and chitosan, collectively known as chitooligosaccharides (COS), have been widely utilized for in vitro experiments, which have identified several signaling pathways affected by chitin. COS have been implicated both in stimulating the immune system itself and in shaping the immune responses to other PAMPs, in particular lipopolysaccharide (LPS) (Liu et al. 2010; Ma et al. 2011; Li et al. 2014). Like the particulate chitins, COS have been attributed both pro- and anti-inflammatory effects. Whether pro- or anti-inflammatory, the outcome seems to be mediated through effects elicited on mitogen-activated protein kinase (MAPK) signaling cascades and on nuclear factor kappa B (NF-κB) signaling (Lin et al. 2007; Wu and Tsai 2007; Liu et al. 2010).

A major factor affecting the nature of the immunostimulating effects of COS on macrophages is prior stimulation by interferon gamma (IFN-γ). In macrophages, that have been primed by previous stimulation by IFN-γ, a general proinflammatory effect of COS stimulation is manifested as TNF-α expression and elevated nitric oxide production through increased NF-κB nuclear translocation (Seo et al. 2000; Wu and Tsai 2007). In contrast, COS pretreatment attenuated the MAPK signaling and NF-κB-mediated inflammatory signaling induced by LPS treatment in macrophages (Ma et al. 2011) and human umbilical vein endothelial cells (Liu et al. 2010). Recently, a new report concluded that COS can inhibit the LPS-induced O-GlcNAc-ylation of NF-κB subunit p65 and impede nuclear translocation and NF-κB DNA binding directly (Li et al. 2014). Apparently, the immunostimulatory effects of COS are rather complex and is dependent upon other factors such as IFN-γ.

Overall, the emerging picture indicates that very short COS exert proinflammatory stimulation on IFNγ primed macrophages (Wu and Tsai 2007), while COS pretreatment of macrophages attenuates the inflammatory signaling responses to other PAMPs such as LPS (Ma et al. 2011) and to proinflammatory cytokines such as TNF-α (Lin et al. 2007).

Chitin is not unique among glycans in exerting immune stimulation in a size and tissue-dependent manner, several glycosaminoglucans exhibit similar properties. Especially in the case of hyaluronic acid (HA) (see Figure 1 for structure), the analogy is appropriate. Short fragments of HA have been found to induce a proinflammatory response in macrophages (McKee et al. 1997), a function that is dependent on TLR-2 and -4 and has been confirmed in several tissues, including the lung (Jiang et al. 2005) and peritoneum (Zheng et al. 2009). Very short fragments of HA have been found to induce vasculogenesis in endothelial cells and angiogenesis (West et al. 1985; West and Kumar 1989). Polymeric high-molecular-weight HA in contrast exhibits anti-inflammatory properties by maintaining regulatory T-cell populations and inducing IL-10 expression (Bollyky et al. 2009, 2011). Furthermore, high-molecular-weight HA exerts anti-angiogenic properties, which are thought to stem from displacement of HA fragments from cellular receptors by the bulky and water absorbing polymeric HA (Deed et al. 1997).

### Receptor interactions

The complexity of chitin-mediated inflammatory signaling may stem from the differing abilities of chitins of different chain lengths and particle sizes to stimulate a broad range of receptors. This in turn leads to differences in effects due to different receptor interplay in various tissues, and problems in reconciling in vivo observations with those made in vitro. The pattern recognition receptors TLR-2 (Da Silva et al. 2008, 2009), TLR-9 and nucleotide-binding oligomerization domain-containing protein 2 (NOD-2) (Wagener et al. 2014), mannose receptor (Shibata et al. 1997; Da Silva et al. 2009) and dectin-1 (Da Silva et al. 2009; Mora-Montes et al. 2011) and the chemoattractant receptor-homologous molecule expressed on Th2 cells (Kogiso et al. 2011) have all been implicated in chitin signaling in different, but often overlapping, cellular subsets. Each receptor impacts different elements of chitin signaling, and studies have found that chitin influences the cellular expression of at least some of the receptors involved in the chitin signaling (Kogiso et al. 2011; Koller et al. 2011).

### Developmental functions of chitins

The ability of COS to shape the development and morphological patterning in vertebrates has been explored in zebrafish. The investigation into their function in development was initiated due to the observation that hyaluronan synthase 2 (*HAS2*), which is found, among others, in zebrafish and mice, synthesizes short chitin oligomers during certain stages of zebrafish development (Semino et al. 1996). Observations that intracellular injections of bacterial chitinases and extracellular injections of allosamidin, a substrate analog inhibitor of chitinases and chitinase-like proteins (CLPs), lead to similar developmental phenotypes characterized by severely impeded trunk and tail formation (Semino and Allende 2000) seem to indicate that COS may impact zebrafish development and morphological patterning in a compartmentalized fashion. The allosamidin sensitive agent involved is likely to be a chitinase or CLP, and the zebrafish CHIA.4 was proposed as a candidate (Koch et al. 2014). At present we have only limited functional understanding of these findings, but observations seem to point to effects exerted via MAPK signaling. Chitin tetramers have been shown to activate MAPK members extracellular signal-regulated kinase 1 (ERK-1) and -2 in zebrafish embryonic cell lines (Snaar-Jagalska et al. 2003). Interestingly, the morphological consequence of morpholino knockdown of either ERK-1 or -2 in zebrafish embryos (Krens et al. 2008) resembles those resulting from disturbing COS and chitinase/CLP equilibrium, with impaired trunk and tail formation (Semino and Allende 2000).

**Fig. 3.**
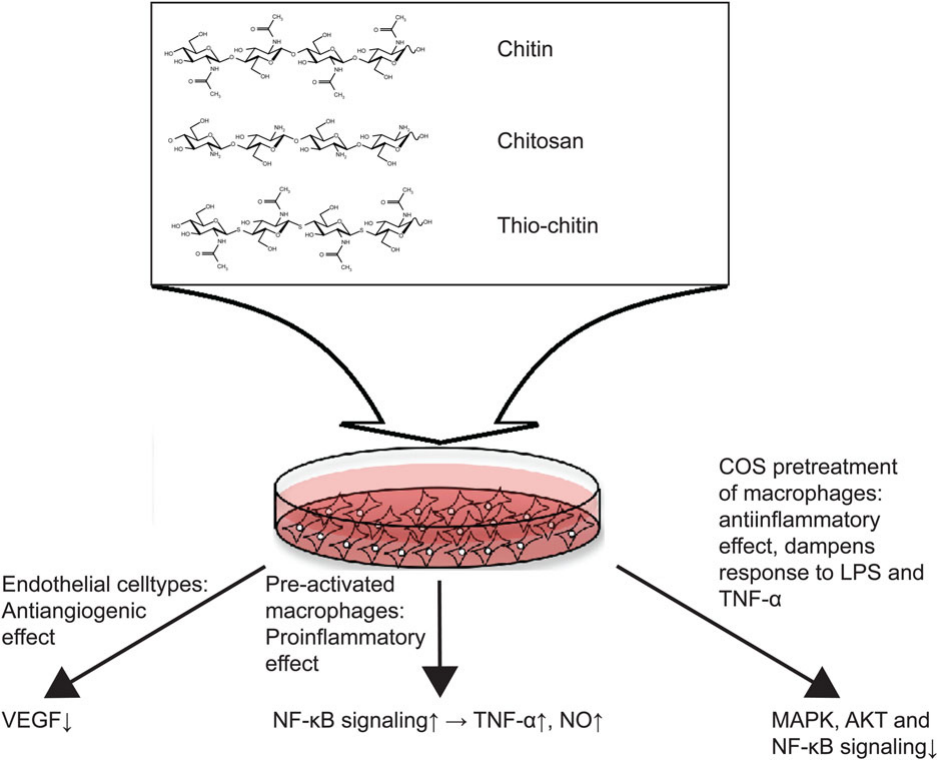
In vitro observations of COS signaling functions. Oligomeric soluble chitin, chitosan and unhydrolyzable thio-chitin have been tested in various cellular systems to investigate the molecular signaling pathways affected. In endothelial cell-types COS fractions have been observed to exert anti-angiogenic effects which can to some extent be accounted for by downregulation of VEGF (Xiong et al. 2009). In macrophages, the outcome of COS stimulation is dependent on prior stimulations: in IFN-γ primed macrophages COS stimulation has a proinflammatiory effect by enhancing NF-κB signaling (Wu and Tsai 2007), while pretreatment of macrophages with COS dampens inflammatory effects of other stimuli such as LPS, by blocking MAPK, AKT and NF-κB signals (Ma et al. 2011).

COS have also been found to inhibit angiogenesis in several different experimental settings, both in healthy development and in cancer. Effects have been observed both ex vivo by the chorioallantoic membrane (CAM) vessel development assay and in vivo in zebrafish (Wang et al. 2007). Utilizing the CAM assay, the effective fractions have been dissected in terms of degree of polymerization and degrees of acetylation of the COS fractions (Wu et al. 2012) and by qPCR several candidate genes have been found to be differentially expressed upon COS stimulation (Xiong et al. 2009) (see Figure 3 for a summary of the observed effects of COS stimulation in different cellular systems).

### Applications in bioengineering

Chitosan, being more soluble than chitin and exhibiting many possibilities for functionalization, has found wide industrial and pharmaceutical applications, e.g. in wound dressings and hydrogels (see Kumar et al. 2004 for an extensive review). In recent years, these applications have been further refined in bioengineering for model organs and tissue replacement therapies, serving as an artificial extracellular matrix allowing for specific patterning to induce, e.g. stem cell differentiation (Gerberich and Bhatia 2013). By such means, the engineering of cellular systems has developed into an exciting emerging field increasing rapidly in complexity, and providing enhanced model systems for the research into cellular communication, proliferation, etc. (see Gnavi et al. 2013 for a recent review of chitosan in bioengineering applications).

## Chitinases

The effects of particle size, reflecting the degree of polymerization, of chitinous stimulants for their impact on vertebrate biology suggests that proteins binding and/or hydrolyzing chitin are mediating and shaping these effects. In higher vertebrates, chitinases and CLPs contribute to such functions. On the basis of amino acid sequence similarities, chitinases are classified into two distinct families, the glycoside hydrolase families 18 and 19 (GH18 and GH19). Vertebrate chitinases all belong to the GH18 family, which is evolutionarily ancient with members found in species from all kingdoms of life. The GH18 family is very diverse; it comprises variable numbers of members within different vertebrate species and includes different enzyme activities, including endo- and exochitinases (Horn et al. 2006), as well as non-hydrolytically active proteins known as CLPs or chitolectins. In addition to these, the GH18 also includes enzymes with specificities for other GlcNAc-containing structures, e.g. peptidoglycan and mammalian glycoconjugates (Bokma et al. 1997; Larsen et al. 2014).

### Evolution of vertebrate chitinases

The evolution of the GH18 family has been analyzed and reviewed in a number of studies (Bussink et al. 2007; Funkhouser and Aronson 2007; Hussain and Wilson 2013). The GH18 family members are separated into three major phylogenic clades: (i) the hydrolytically non-active chitinase domain-containing proteins (CHID), (ii) the exoacting hydrolases named chitobiases (CTBS) and (iii) a multimembered group containing both chitinolytically active endochitinases and hydrolytically inactive CLPs (Funkhouser and Aronson 2007). The former two are represented by one member per species, while the chitinase/CLP group has undergone an extensive expansion in vertebrates due to several gene duplications. In mammals, the chitinase/CLP group invariably includes two active chitinases, the chitotriosidase (CHIT1) and the acidic chitinase (CHIA), in addition to a variable number of CLPs, among others the chitinase 3-like 1 (CHI3L1) (Bussink et al. 2007). An ancient gene duplication giving rise to the two active endochitinases CHIT1 and CHIA, both of which are found in all mammals, is believed to have taken place sometime between the emergence of jawless and jawed fish (Hussain and Wilson 2013). Further duplications and loss-of-function mutations have later led to the emergence of several CLPs, some of which are found in all mammals, while others are specific to particular species. As an example, the rodent genomes encode four CLPs that are absent in non-rodents (Bussink et al. 2007; Hussain and Wilson 2013).

**Fig. 4.**
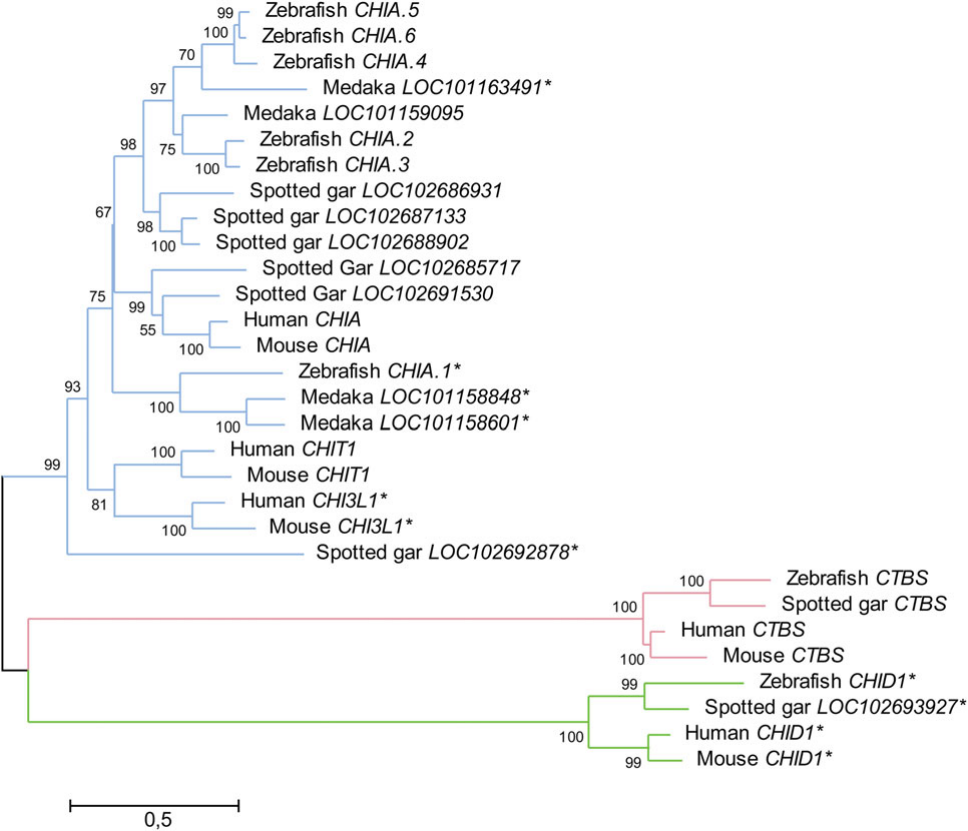
Phylogenetic tree of GH18 domains from three fish species, with selected human and mice genes. The overall phylogeny is characterized by one *CHID1* and *CTBS* gene per species, and an expanded group of chitinase/CLP-encoding genes. Most of the zebrafish genes, *CHIA.2* to *CHIA.6*, are placed in the *CHIA* superclade of the chitinase/CLP phylum. As also suggested by Hussain and Wilson (2013), it seems that, if indeed any zebrafish members of the *CHIT1* superclade within the chitinase/CLP phylum exist, *CHIA.1* seems the most likely candidate. Sequences with mutations in the active DxxDxDxE motif are indicated with an asterisk. Method: Nucleotide sequences encoding the GH18 domain of each of the genes were aligned by ClustalW. The best substitution model, as measured by the lowest Bayesian information criterion score, was found to be the general time reversible with five discrete gamma distributions and assuming the presence of invariable sites. The phylogeny was inferred by maximum likelihood, and 500 bootstrap replications were applied to test the phylogeny. The percentage bootstrap values are given next to each branchpoint. The evolutionary analysis was performed using MEGA6 (Tamura et al. 2013).

The three major clades of GH18 proteins can be recognized in all vertebrates. However, the expansion of the chitinase/CLP group makes it harder to assign one-to-one orthologs between more distantly related species (see Figure 4). In fish genomes, there is a general propensity to encode a higher number of proteins that, by virtue of conserved active amino acid motifs and retention of chitin-binding domains, are predicted to encode active chitinases. The zebrafish genome, for example, encodes five predicted active chitinases and one predicted CLP. The emergence of the high number of genes has been attributed to a whole-genome duplication (WGD) specific to teleost fish (Hussain and Wilson 2013). However, mining the genome of the spotted gar (*Lepisosteus oculatus*), which branched off the teleost linage before the WGD (Amores et al. 2011), we have identified five genes encoding predicted active chitinases and one predicted to encode a CLP, the same number of predicted active chitinases in the chitinase/CLP group as in zebrafish (see Table I). This indicates that the number of genes has normalized itself after the WGD event.

**Table I.** One of the clearest differences between the development of GH18 family members in mammalian and fish genomes is that the number of predicted active endochitinolytic members of the chitinase/CLP group relative to that of the CLPs

Species	Phylogenetic group		Activity
Human	Chitinase/CLP CTBS CHID	6 1 1	2 endo/4 CLP exo no
Mouse	Chitinase/CLP CTBS CHID	8 1 1	2 endo/6 CLP exo no
Zebrafish	Chitinase/CLP CTBS CHID	6 1 1	5 endo/1 CLP exo no
Spotted Gar	Chitinase/CLP CTBS CHID	6 1 1	5 endo/1 CLP exo no
Medaka	Chitinase/CLP CTBS CHID	5 1 1	4 endo*/1 CLP exo no

Mammalian genomes invariably encode two active endochitinases, whereas most of the fish genomes encode 3–5 genes encoding proteins predicted to have retained their hydrolytic abilities, based on the conservation of the active motif. Medaka (*Oryzias latipes*) is an exception to this rule.

The active GH18 domain, a well-conserved triosephosphate isomerase (TIM) barrel fold, constitutes the defining feature of the GH18 family. In the active site, three aspartate residues and a glutamate residue form an all-important DxxDxDxE motif, where the Glu residue functions as the catalytic proton donor. The nonhydrolytic GH18 proteins are believed to have lost their hydrolytic capacities due to substitutions in this position. The active endochitinases furthermore exhibit a flanking chitin-binding domain, which is absent in CLPs, CHIDs and CTBSs.

### Human and murine chitinases and CLPs

The genes and proteins of the GH18 family have primarily been studied in humans and mice, and these studies have elucidated their wide-ranging functions in immunity and tissue homeostasis. Here, we will briefly introduce the most important members, which are found in all mammals.

Chitinase 1 (CHIT1), also known as chitotriosidase, was the first active chitinase to be discovered in humans (Boot et al. 1995). The CHIT1 gene is expressed in various tissues and CHIT1 is the main functional chitinase in the human lung (Seibold et al. 2008). The expression is primarily derived from activated macrophages and neutrophils (Boot et al. 1995; Boussac and Garin 2000; Malaguarnera et al. 2006). CHIT1 is expressed in response to various proinflammatory cues in a complementary fashion in neutrophils and macrophages. TLR signaling is a potent inducer in neutrophils, while NOD-2 signaling induces *CHIT1* in macrophages (van Eijk et al. 2007).

CHIA, the second active endochitinase in the GH18 family, is named after its unusually low pH optimum of activity and is encoded by the *CHIA* gene (Boot et al. 2001). In humans, *CHIA* is primarily expressed in the liver, lungs, heart and thyroid gland, whereas in mice the gene is most highly expressed in the stomach (Ohno et al. 2013).

Chitinase 3-like 1 (Chi3L1) is a CLP and has no chitinolytic activity. Chi3L1 was first described in bovine mammary tissue (Rejman and Hurley 1988). Through extensive carbohydrate (Shackelton et al. 1995; Fusetti et al. 2003; Houston et al. 2003) and protein (Bigg et al. 2006; He et al. 2013) binding capacities it is able to interact with several different receptors and impact cell proliferation (De Ceuninck et al. 2001; Recklies et al. 2002) and cell survival (Chen et al. 2011; Francescone et al. 2011). Due to these functions, much attention has been focused on Chi3L1 in immunological and cancer research.

Oviductal glycoprotein 1 (OVGP1), as the name suggests, has been found expressed in oviduct and zona pelucida of the post-ovulatory oocytes (McBride et al. 2004). It has been suggested to enhance fertilization by interacting with a specific protein on capacitated sperm (Kadam et al. 2006).

Chitobiase (CTBS) is the only exoacting enzyme of the vertebrate GH18 family and is known to be of importance to lysosomal glycoprotein turnover (Aronson et al. 1989; Persichetti et al. 2012).

Chitinase domain-containing 1 (CHID1) is a CLP since it has no chitin hydrolytic activity. It is clearly evolutionarily distinct from the chitinase/CLP group of genes and proteins and is not included in this group. The gene is expressed and the protein secreted by alternatively activated macrophages (Kzhyshkowska et al. 2006). CHID1 has been demonstrated to bind to LPS and induce inflammatory cytokine production from macrophages in vitro (Meng et al. 2010).

### Chitinases in immunity and disease

Since the first discovery of active chitinases in mammals, the importance of chitinases in relation to mammalian diseases has been recognized. In fact, the discovery of a marked elevation of chitinolytic activity in plasma from patients suffering from the lysosomal storage disorder Gauches disease (Hollak et al. 1994) preceded the discovery of the responsible *CHIT1* gene, which encodes the first active chitinase to be identified in humans (Boot et al. 1995).

Following this discovery, several members of the chitinase/CLP group have been recognized for their role in mediating and directing immune responses; most often to the effect of directing an IL-13 driven TH2 response. The functions of chitinases and CLPs have been studied in several allergic and autoimmune disease models as well as in infection and models of cancer development. See Table II for an overview of mammalian chitinases with reference to known disease involvements.

**Table II.** Overview of GH18 family members encoded in the human genome, their molecular function and disease implications

HGNC symbol	Species	Molecular functions	Disease involvement	Key references
CHIT1	All mammals	Endochitinolytic activity, hydrolysis of GlcNAc containing glycosides, e.g. LacDiNAc	Asthma, fungal infection, *H. pylori* infection, Gauchers disease and sarcoidosis	Hollak et al. (1994), van Eijk et al. (2005), Gavala et al. (2013), Lee et al. (2012),Cozzarini et al. (2009)
CHIA	All mammals	Endochitinolytic activity, stimulate PI3K and AKT signaling	Asthma and nematode infection	Boot et al. (2001), Zhu et al. (2004), Hartl et al. (2009), Nance et al. (2012)
CHI3L1	All mammals	Glycoside and protein binding, mediate IL-13 signaling, stimulate MAPK signaling	Asthma, fibrosis, cancer, intestinal inflammation and bacterial infection	Houston et al. (2003), He et al. (2013), Lee et al. (2009), Tang et al. (2013), Francescone et al. (2014), Tran et al. (2014), Dela Cruz et al. (2012), Shao et al. (2009)
OVGP1	All mammals	Protein binding	Suggested as a marker of ovarian cancer	Kadam et al. (2006)
Chil2	Absent in rodents	Glycoside binding, stimulate MAPK signaling	Arthritis	Areshkov et al. (2011), Steck et al. (2002), Miyatake et al. (2013)
CTBS	All mammals	Exochitinolytic activity, lysosomal glycoprotein turnover	None	Aronson et al. (1989), Persichetti et al. (2012)
CHID1	All mammals	Glycoside binding, stimulate MAPK signaling	Arthritis	Kzhyshkowska et al. (2006), Xiao et al. (2014)

Orthologs of the genes encoding the two active endochitinases, CHIA and CHIT1, the non-hydrolytical CHI3L1, OVGP1 and CHID1, as well as the exoacting CTBS, are found in all mammals. The CLP-encoding Chil2 is absent in rodents, which in exchange feature four rodent-specific CLP-encoding genes (Chil3, Chil4, Chil5 and Chil6).

### Protective roles of active chitinases against chitin-producing pathogens

The two active mammalian chitinases, the CHIT1 and CHIA, confer protection against chitin-producing invading pathogens. Both enzymes degrade chitin-containing protective structures and release of degradation products is assumed to induce innate immunity. When recombinantly expressed and injected intraperitoneally in mice, the human CHIT1 decreases mortality from fungal infection (van Eijk et al. 2005). The fungal-derived β-glucan curdlan potently induces CHIT1 in human phagocytes through stimulation of dectin-1 (van Eijk et al. 2010). Chitin, which has also been demonstrated to confer inflammatory signaling through dectin-1 (Da Silva et al. 2009), might also be expected to induce *CHIT1* expression. This has not been shown directly, but it has been demonstrated that fungal cell wall derived β-glucan and chitin act synergistically in murine lungs. Applied together they exhibited stronger immunogenic properties and induced higher levels of chitinase activity than either did on their own. However, which specific chitinase was responsible for the increased chitinase activity was never tested (Dubey et al. 2014).

Likewise, CHIA has been found to be important for direct protection against chitinous invaders and is potently induced by helminth infections in mouse lungs (Reese et al. 2007). In mouse brains, CHIA released from alternatively activated macrophages was found to be of vital importance for lysis of tissue cysts formed by the parasitic nematode *Toxoplasma gondii*. Mutants deficient in CHIA displayed impeded parasite eradication and ultimately lower survival rates to infection (Nance et al. 2012).

### Alveolar hyperresponsiveness, asthma and fibrosis

The discovery of a CHIA implication in the pathology of asthma in a murine ovalbumin (OVA) challenged asthma model has attracted considerable attention. In this model, CHIA was strongly induced by OVA challenge in an IL-13-dependent manner, and was furthermore found to be pivotal to further induction of IL-13-driven TH2 inflammation and immune cell accumulation (Zhu et al. 2004). Interestingly, while CHIA is central to OVA-induced (non-chitinous) allergic reactions, studies employing CHIA overproducing mouse lines and CHIA pretreatment of chitinous stimulants found that CHIA confers significant protection against chitin-induced alveolar inflammation (Reese et al. 2007). CHIA have also been found to serve an important role in protecting airway epithelial cells from undergoing apoptosis by stimulating phosphoinositide 3-kinase (PI3K) and AKT signaling, through a mechanism which appears to be related to the chitin-binding site, but independent of chitinolytic capacity of the protein (Hartl et al. 2009).

More recently, CHIT1 have also been implicated in human airway hyperresponsiveness and asthma (Gavala et al. 2013), as well as being instrumental to IL-13-driven alveolar fibrosis by augmenting transforming growth factor beta (TGF-β) and MAPK signaling in mice (Lee et al. 2012). CHIA and CHIT1 are not the only GH18 proteins with a role to play in pathologic TH2 inflammation. By a very comprehensive in vivo study using several KO mouse lines, it was shown that Chi3L1 is central to the TH2 dominated inflammatory responses to both chitinous and non-chitinous (OVA) challenges, including the initial IL-13-dependent induction of CHIA and alternative macrophage activation. Type 2 innate immunity and TH2 responses were largely impeded in Chi3L1 KO mice (Lee et al. 2009). A more profound understanding of many of the immunomodulatory functions of CHI3L1 has long been hampered by the lack of identification of a receptor that could mediate the signaling. A major breakthrough in the understanding of many of the CHI3L1 signaling properties was the revelation that CHI3L1 binds to the interleukin 13 receptor alpha 2 (IL-13Rα2), in concert with IL-13, to activate MAPK and Wnt signaling pathways. These studies have established Chi3L1 as a key component in most IL-13-driven immune responses, as well as in protecting immune cells from apoptosis and pyroptosis (He et al. 2013).

### Intestinal inflammation and bacterial infections

Chitinases and CLPs, especially Chi3L1, have been studied for their involvement in intestinal inflammation and different pathologies involving the integrity of the mucosal barriers of the stomach and gastrointestinal tract such as inflammatory bowel disorders.

A significant correlation has been shown between expression of *CHIT1* in gastric mucosa and *Helicobacter pylori* infection (Cozzarini et al. 2009). As yet no functional data verify a direct role for CHIT1 in *H. pylori* infection or gastrointestinal pathology. However, the GlcNAc containing glycoprotein decoration LacDiNAc, found on gastric mucins and hypothesized to negatively impact *H. pylori* adhesion to the gastric mucosa (Kenny et al. 2012), was recently found to constitute a substrate for CHIT1 hydrolysis at turnover rates comparable with those of the native substrate (Larsen et al. 2014). The possible involvement of auxiliary substrate specificities of CHIT1 in pathogen adherence is interesting, but requires further experimental tests. Following another line of investigation it was recently shown that downregulation of CHIA gene expression was strongly correlated with corpus atrophy in *H. pylori* infection. The observation of loss of *CHIA* expression was therefore suggested as a clinical marker of corpus atrophy (Nookaew et al. 2013).

It has long been known that *Chi3L1* is specifically upregulated in inflammatory conditions of the gut, and infection studies have suggested a function in both development and resolution of intestinal inflammation as well as bacterial clearance. The Chi3L1 gene is strongly expressed in inflamed colonic mucosa and enhances *Escherichia coli*, and *Salmonella enterica* serovar *typhimurium* infection (Mizoguchi 2006). The infection promoting effects have been found to stem from enhanced adhesion of bacteria to intestinal epithelial cells (IECs) (Kawada et al. 2008), specifically through bacterial interaction with N-glycosylation patterns on Chi3L1 expressed by IECs (Low et al. 2013). However, Chi3L1 also promotes clearing and resolution of bacterial infections and inflammation in colitis through Stat3 signaling (Tran et al. 2014). Therefore, the role of Chi3L1 in intestinal inflammation and normal homeostasis is not entirely clear and this issue needs additional investigation.

Also, Gram-positive bacterial infections have been associated with Chi3L1. A correlation between elevated serum levels of Chi3L1 and infection with *Streptococcus pneumoniae* was reported >10 years ago (Kronborg et al. 2002). More recently, a comprehensive study utilizing Chi3L1 KO mouse lines and intratrancheal bacterial infections explored the functional role. Chi3L1 confers a marked protection against *S. pneumoniae* infection, enhancing the ability of macrophages to kill bacteria and at the same time protecting the immune cells from pyroptosis by inhibiting IL-1β-driven inflammosome activation (Dela Cruz et al. 2012).

### Chitinases and CLPs in cancer

The Chi3L1 is an important regulator of cellular survival, proliferative signaling and angiogenesis in a number of different cells and tissues, and it has been studied with interest for its ability to promote a range of different oncogenic signaling events. Among the best-characterized functions are the mediation of integrin/focal adhesion kinase (FAK) signaling leading to enhanced angiogenesis through MAPK signaling and the release of vascular endothelial growth factor (VEGF) in brain cancers. Furthermore, Chi3L1 induces increased radio resistance through AKT signaling (Shao et al. 2009; Francescone et al. 2011). In addition to the induction of vascular formation, CHI3L1 also contributes to the stability and integrity of newly formed vessels by enhancing intercellular contacts between mural cells and endothelial cells forming the vessel wall (Francescone et al. 2014). Chi3L1 has also been found to promote colorectal cancer development via the induction of MAPK signaling leading to cellular proliferation and angiogenesis (Kawada et al. 2012).Through induction of several inflammatory factors, Chi3L1 has been proposed to enhance inflammation mediated metastasis of breast cancer in xenografted tumor bearing mice (Libreros et al. 2012).

### Other substrates and functions for vertebrate GH18 family members

GH18 proteins have in some instances been found to display a certain functional plasticity, e.g. CHIT1 displays transglycosidase activity under certain conditions (Aguilera et al. 2003). Furthermore, some GH18 family members hydrolyze other GlcNAc containing substrates such as peptidoglycan (Bokma et al. 1997).

The GlcNAc containing glycoprotein decoration LacDiNAc is a substrate for CHIT1 (Larsen et al. 2014). Interestingly, Stat3 signaling in mouse embryonic stem cells is regulated by the presence of LacDiNAc glycosyl decoration on the leukemia inhibitory factor receptor. Absence of LacDiNAc leads to the cessation of self-renewal of the stem cells (Sasaki et al. 2011), indicating that accessory hydrolytic functions of CHIT1 might be of importance to cellular signaling. However, such in vivo functions remain to be shown experimentally. LacDiNAc has also been described as an O-linked glycoprotein decoration of the zona pellucida glycoprotein 3, which is regarded as important to sperm binding to the egg at the initiation of fertilization (Dell et al. 2003). Another GH18 family member present in all mammals, the OVGP1, is expressed in the zona pellucida of post-ovulation oocytes in golden hamster (McBride et al. 2004) and is thought to be an important facilitator of fertilization via interactions with non-muscle myosin present on capacitated sperm (Kadam et al. 2006). Whether OVGP1 will bind to LacDiNAc remains to be established, but the possibility merits further attention.

Though not an active chitinase, Chi3L1 has retained the ability to bind chitin of various chain lengths (Fusetti et al. 2003; Houston et al. 2003), and it has been suggested that conformational changes of the protein surface structure may be a part of the functional mechanism (Houston et al. 2003). In addition to the binding of various chitins, Chi3L1 also binds heparin (Shackelton et al. 1995), and collagen types I, II and III (Bigg et al. 2006). At least some of the multiple signaling properties of Chi3L1 stem from its ability to bind to both glycosides and proteins. For example, heparin decorations on Syndecan 1 (Syn1) are necessary for Chi3L1-mediated Syn1 interaction with the integrin αvβ3, which leads to FAK signaling and ERK1/2 activation, which promotes tumor angiogenesis (Shao et al. 2009).

### Chitin and chitinases in disease and health

The biological pathways affected by chitin stimulation in vertebrates overlap extensively with those affected by members of the chitinase/CLP group of vertebrate GH18 proteins. However, the possible direct interactions between chitin and chitinase/CLP-mediated signaling have only rarely been addressed.

In one such study, many of the proangiogenic and metastasis promoting effects attributed to elevated Chi3L1 were counteracted by a repeated IP CMP delivery regimen. This treatment ultimately led to substantially reduced tumor growth and metastasis, indicating that chitin can impede Chi3L1 signaling (Libreros et al. 2012). However, it is not known by which mechanism this effect is achieved. Other studies have shown that COS can stimulate Chi3L1 functions, e.g. the Chi3L1-induced proliferation of human osteoarthritic chondrocytes was markedly stimulated by COS in vitro (Einarsson et al. 2013).

Several of the exciting new discoveries over the last few years have provided hints of possible interactions which deserve further experimental exploration. The epithelial-derived cytokine signaling induced by chitin, the importance of which is becoming increasingly appreciated, provides several good examples. One of several signaling events described to lead to alternative macrophage activation in response to CMP challenge in murine lungs is CCL2 release from epithelial cells (Roy et al. 2012). CHIA, which is known to be released in response to alveolar infection with chitinous pathogens such as helminths (Reese et al. 2007), induces the release of CCL2 in mouse lungs, this is one of the functions of CHIA which is not dependent on chitinolytical activity (Hartl et al. 2009). It seems very likely that there is a link between these stimuli which both lead to CCL2 release in lung tissue, but at present it is not known how they connect. In the original paper describing human CHIA, it was shown that this enzyme retains <20 pct. of its chitinolytic activity at pH 7 (Boot et al. 2001). It is tempting to speculate that CHIA, along with its well-documented IL-13-mediating functions (Zhu et al. 2004), might be responsible for the release of CCL2 described by Roy et al. (2012), possibly by binding to chitin directly. Likewise, the epithelial-associated cytokine TSLP, recently reported to contribute to TH2 inflammatory signaling mediated by ILC2s in response to chitin challenge in the murine lung (Van Dyken et al. 2014), is induced in a Chi3L1-dependent manner in the immune responses to *S. pneumoniae* in murine lungs (Dela Cruz et al. 2012). Again it is tempting to speculate that Chi3L1, which is known to bind chitin (Houston et al. 2003), could be involved in the perception of chitin and the induction of TSLP.

## Chitin and chitinase research in the future

The complex and often opposing responses evoked in vertebrates by chitin stimulations limits the utility of in vitro models with their inherent limitations in mimicking the complexity of actual biological systems. With the advent, in recent years, of increasingly sophisticated multicellular in vitro approaches such as organs-on-a-chip it seems researchers might soon be better equipped to study the effects of chitin and COS in systems more faithfully reproducing the complexity of living systems, and be better able to reconcile diverging experimental observations. Such approaches may be particularly beneficial to the studies of COS regulation of cell proliferation, angiogenesis or cancer (see Young 2013 for a recent review of organ-on-a-chip approaches to cancer developmental research).

**Fig. 5.**
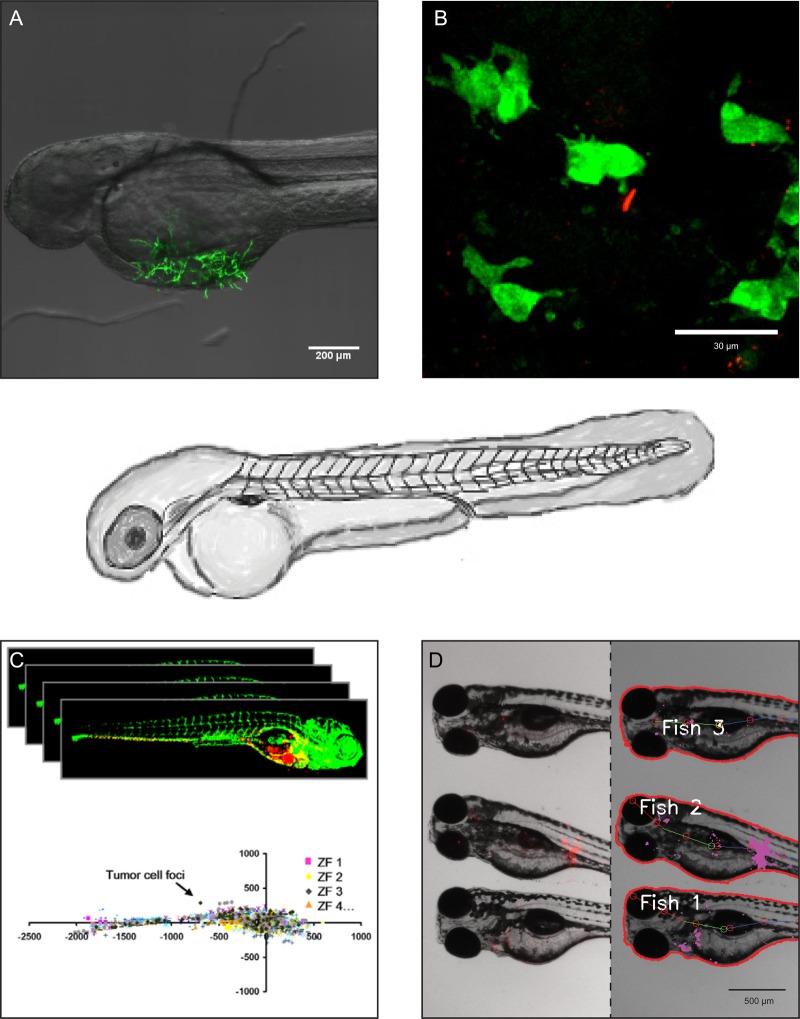
Zebrafish embryos for in vivo investigation of the role of chitinases in vertebrate disease progression. The characteristics of the zebrafish model can be taken advantage of in investigations of molecular and cellular interactions from highly detailed confocal microscopy to high-throughput software-based image analysis. (**A**) 2 DPF zebrafish embryo infected by GFP expressing *Aspergillus niger*. (**B**) High-magnification confocal microscopy image of a zebrafish transiently expressing a microinjected chitinase:eGFP reporter fusion construct driven by the natural promoter of the chitinase gene, and expressed as extrachromosomal DNA. The rod-shaped red fluorescent cell is a *S. typhimurium* cell, which has been injected into the embryo at 28 h post fertilization. (**C**) High-throughput software-assisted confocal image analysis has been applied to study metastatic behavior of injected cancer cells with strong statistics. Image from Ghotra et al. (2012). (**D**) Medium-throughput fluorescent microscopy image analysis can be used to follow progression of bacterial infections by automated pixel-count and image recognition.

The exciting recent findings linking chitinases and CLPs to the cellular immune responses towards bacterial (Cozzarini et al. 2009; Dela Cruz et al. 2012; Tran et al. 2014) and fungal (Wagener et al. 2014) infections highlights the necessity for an amenable in vivo infection model. One such model for studies of disease progression and impact of genetic and pharmaceutical approaches could be the zebrafish. The optical clarity of the developing larvae makes it amenable to real-time evaluation of disease progression and high-throughput compound screening. These unique features allow for high-throughput, as well as detailed, microscopy and image-based analysis of disease progression and innate immune cell response patterns (see Figure 5) and (Brothers et al. 2013; Knox et al. 2014).

One limitation of the zebrafish model in human research is assigning orthology to human genes and proteins. In spite of the obvious evolutionary relationship between the members of the chitinase/CLP groups across species, caution must be observed when trying to conclude functional relationships. In order for the zebrafish to realize its potential as a model of chitinase/CLP biology, a thorough functional and expressional characterization is essential, and initial approaches have commenced. The expression patterns of the six members of the zebrafish chitinase/CLP-encoding group of genes in normal embryonic and early larval development have been described (Koch et al. 2014). One of the genes, *CHIA.3*, was the focus of a recent paper that confirmed the chitinolytic activity of the protein and showed that the recombinant protein inhibits the growth of *Candida albicans* and lowers the mortality rates induced by *Candida* infection (Teng et al. 2014). Another zebrafish chitinase/CLP gene, *CHIA.6*, was found among the most upregulated genes in response to infection with *S. typhimurium* by chip analysis (Stockhammer et al. 2009), hinting that this gene family may be involved in immune responses to bacterial as well as fungal infections in zebrafish as it seems to be in mice.

## Funding

This work was supported by the CARB Centre, the Danish National Research Foundation, Aarhus University, Grant No. DNRF79. Funding for open access charge: CARB Centre, the Danish National Research Foundation, Aarhus University, Grant No. DNRF79.

## Conflict of interest statement

None declared.

## Abbreviations

CAM, chorioallantoic membrane; CHID, chitinase domain-containing proteins; CHID1, chitinase domain-containing 1; CHIT1, chitinase 1; CLPs, chitinase-like proteins; CMPs, chitin microparticle; COS, chitooligosaccharides; CTBS, chitobiases; ERK-1, extracellular signal-regulated kinase 1; FAK, focal adhesion kinase; GlcNAc, *N*-acetyl-d-glucosamine; HA, hyaluronic acid; *HAS2*, hyaluronan synthase 2; IECs, intestinal epithelial cells; IFN-γ, interferon gamma; IL, interleukin; ILC2s, innate lymphoid type 2 cells; IN, intranasally; LPS, lipopolysaccharide; MAPK, mitogen-activated protein kinase; NF-κB, nuclear factor kappa B; NOD-2, nucleotide-binding oligomerization domain-containing protein 2; OVA, ovalbumin; PAMPs, pathogen-associated molecular patterns; PI3K, phosphoinositide 3-kinase; TGF-β, transforming growth factor beta; TIM, triosephosphate isomerase; TLRs, Toll-like receptors; TSLP, thymic stromal lymphopoitin; VEGF, vascular endothelial growth factor**;** WGD, whole-genome duplication.
